# Impact of Portraiture Photography on Orthodontic Treatment: A Systematic Review and Meta-Analysis

**DOI:** 10.7759/cureus.48054

**Published:** 2023-10-31

**Authors:** Mohammad K Alam, Huda Abutayyem, Salah Nazal Alotha, Basant Mousa H Alsiyat, Saif Hamdan K Alanazi, Mashael Hamoud Hammad Alrayes, Raqiyah H Alrayes, Dimah Fayadh Khalaf Alanazi, Haytham Jamil Alswairki, Ahmed Ali Alfawzan, Mohammad Younis Hajeer, Deepti Shrivastava, Kumar Chandan Srivastava

**Affiliations:** 1 Orthodontic Division, Department of Preventive Dentistry, College of Dentistry, Jouf University, Sakaka, SAU; 2 Clinical Sciences, Center of Medical and Bio-Allied Health Sciences Research, College of Dentistry, Ajman University, Ajman, ARE; 3 Orthodontics, School of Dental Sciences, Universiti Sains Malaysia, Kota Bharu, MYS; 4 Preventive Dentistry, College of Dentistry, Qassim University, Ar Rass, SAU; 5 Orthodontics, Faculty of Dentistry, University of Damascus, Damascus, SYR; 6 Periodontics Division, Department of Preventive Dentistry, College of Dentistry, Jouf University, Sakaka, SAU; 7 Department of Oral and Maxillofacial Surgery and Diagnostic Sciences, College of Dentistry, Jouf University, Sakaka, SAU

**Keywords:** aesthetic rehabilitation, digital dentistry, orthodontic appliances, orthodontic treatment, dental photography, portrait photography, digital orthodontics

## Abstract

Due to the clear depiction of facial aesthetics and other craniofacial parameters, portraiture photography (PP) is becoming more and more necessary in modern clinical practice. The studies chosen for this review's inclusion looked at how PP affected the orthodontic treatment and diagnostic procedure on the subjects who were watched in the studies. Studies published within the last decade precisely from 2013 were searched for across major online databases after devising a proper search strategy. Multiple reviewers created a specific data extraction form that was used for the investigation, followed by the evaluation of bias and the variables found in each of the chosen papers. This form was meant for the assessment for various variables encountered in this study. According to the meta-analysis, using PP was related with a statistically significant decrease in the risk of orthodontic treatment and diagnostic modalities, with odds ratios (OR) of 0.52 with a 95% confidence interval (CI) of (0.28, 0.96), and a relative risk (RR) of 0.66 with a CI of (0.45, 0.96). In orthodontics, PP is an important tool that offers useful data for diagnosis, treatment planning, and tracking treatment success. To validate the results of studies like ours, a sizable evidence sample is required due to the limited number of trials that have been performed in this area.

## Introduction and background

Portraiture photography (PP) is the art of capturing images of people, with a focus on their facial expressions, personality traits, and physical features [1]. PP can be done in various settings, such as studios, outdoor locations, or even in the subject's home. The aim of PP is to capture the essence of the person in the photograph, revealing their character, mood, and emotions through the image [2]. The photographer uses lighting, composition, and posing techniques to create a portrait that captures the subject's unique qualities and personality [1].

One of the key elements of PP is lighting. Photographers use various lighting techniques to highlight the subject's best features and create a mood or atmosphere in the portrait [2]. For example, soft, diffused light can create a gentle, dreamy atmosphere, while harsh, directional light can create a more dramatic and intense portrait. Another important aspect is composition. The composition of a portrait can greatly impact its effectiveness in conveying the subject's character and mood [2]. The photographer may use framing, posing, and background elements to create a visually compelling portrait that draws the viewer's attention to the subject. In addition to technical elements, PP also relies heavily on the relationship between the photographer and the subject. A skilled portrait photographer must be able to communicate effectively with their subject to make them feel comfortable and at ease during the photoshoot. This helps to create a relaxed and natural expression in the subject, resulting in a more authentic and compelling portrait [1].

PP typically involves the use of digital cameras, specialized lighting equipment, and a standardized photographic protocol to capture standardized images of the patient's face and teeth from different angles [3]. These photographs can be used for various purposes, including orthodontic diagnosis, treatment planning, and monitoring treatment progress. It is a valuable tool in the medical field, especially in orthodontics, where it plays a crucial role in diagnosis, treatment planning, and monitoring treatment progress [3-5]. PP involves the capture of standardized photographs of a patient's face and teeth using digital cameras and specialized lighting equipment [6]. PP provides detailed information about dental and skeletal relationships, facial asymmetries, and other relevant features that are critical for accurate diagnosis and treatment planning [7]. Orthodontists use PP to assess the patient's current condition and create a customized treatment plan that addresses their specific needs. PP also allows orthodontists to monitor treatment progress and adjust as needed [8].

In orthodontic diagnosis, PP can provide essential information about the size, shape, and position of teeth and jaws, as well as any asymmetries or abnormalities in facial features [8]. PP can also aid in the assessment of the patient's facial aesthetics, which is an important consideration in orthodontic treatment planning. In treatment planning, PP can help orthodontists visualize the patient's current condition and create a treatment plan tailored to their specific needs [9]. PP can also help orthodontists monitor the progress of treatment and adjust as needed. Orthodontic treatment and diagnosis often require precise measurements of dental and facial structures to ensure optimal treatment outcomes. In recent years, this technique has emerged as a potential tool to aid in orthodontic diagnosis and treatment planning [6]. PP involves capturing high-quality photographs of patients' faces and teeth, which can provide valuable information regarding dental and skeletal relationships, facial asymmetries, and other relevant features [10].

Despite the potential benefits of PP in orthodontic practice, there is limited consensus on its efficacy and impact on treatment outcomes. Several studies have examined the use of PP in orthodontic diagnosis and treatment planning, but the results have been inconsistent, and there is a need for a comprehensive review of the available literature [8-10]. Therefore, the aim of this systematic review and meta-analysis is to evaluate the impact of PP on orthodontic treatment and diagnostic protocols. The studies selected for inclusion in this review will examine the impact of PP on the orthodontic treatment and diagnostic protocol on the people who were observed in the selected studies. This review will provide an up-to-date synthesis of the available evidence on the use of PP in orthodontic practice and help to inform clinical decision-making regarding its use in routine practice.

This systematic review and meta-analysis intended to assess how PP affected orthodontic treatment and diagnostic procedures and its impact in the field of orthodontics.

This article was previously posted to the medRxiv preprint server on April 27, 2020.

## Review

Methods

Search Strategy Initiation

**Figure 1:**
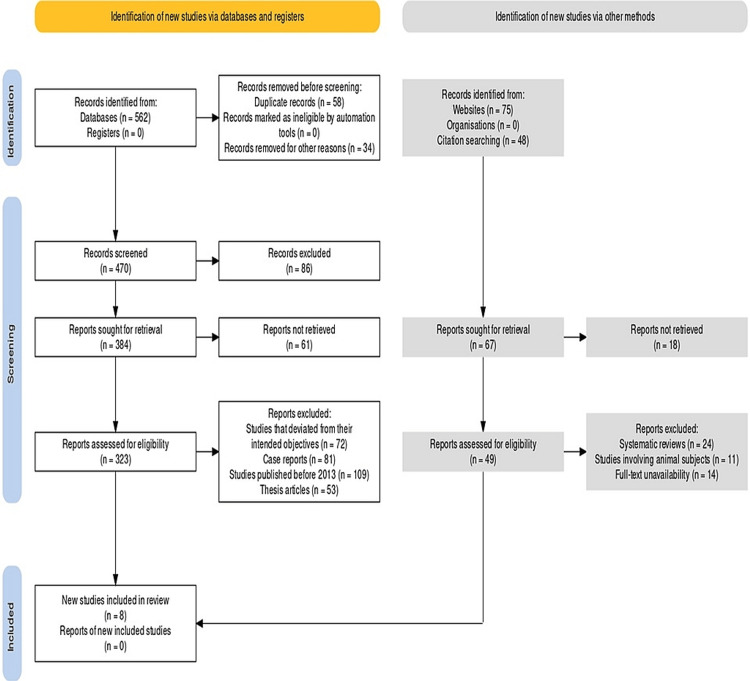
PRISMA protocol PRISMA: Preferred Reporting Items for Systematic Reviews and Meta-Analyses

In accordance with the Preferred Reporting Items for Systematic Reviews and Meta-Analyses (PRISMA) guidelines, registration was completed prior to the commencement of the study (Figure 1) [11]. The research protocol was developed to achieve the objectives and correctly submitted to PROSPERO (CRD42023407261). PICOS is an acronym used in evidence-based medicine to define the key components of a research question. In the case of a systematic review and meta-analysis assessing the impact of PP on orthodontic treatment, the PICOS strategy would be as follows: P - Population: Patients undergoing orthodontic treatment; I - Intervention: PP; C - Comparison: No PP or alternative methods of dental photography; O - Outcome: Improvement in treatment planning, patient education, or treatment outcomes; S - Study design: Randomized controlled trials or observational studies.

Using this PICOS strategy, the research question for this systematic review and meta-analysis was: In patients undergoing orthodontic treatment, what is the impact of PP compared to no PP or alternative methods of dental photography on treatment planning, patient education, and treatment outcomes? This review included randomized controlled trials and observational studies that assess the use of PP in orthodontic treatment and would focus on the impact of this intervention on key outcomes such as treatment planning, patient education, and treatment outcomes.

Search Strategy Implementation

The following search strategy was used across four online databases to identify relevant articles for our investigation using Medical Subject Headings (MeSH) keywords and a combination of Boolean operators:

PubMed- (((((((((((((("Photography"[Mesh]) OR "Portraits as Topic"[Mesh]) OR "Dental Photography"[Mesh]) OR "Orthodontics"[Mesh]) OR "Dentistry"[Mesh]) OR "Dental Care"[Mesh]) OR "Orthodontic Appliances"[Mesh]) OR "Orthodontic Brackets"[Mesh]) OR "Orthodontic Wires"[Mesh]) OR "Orthodontic Records"[Mesh]) OR "Orthodontic Models"[Mesh]) AND (("Treatment Outcome"[Mesh]) OR "Patient Education as Topic"[Mesh])) AND (((((((("Humans"[Mesh]) AND "English"[lang]) AND ("2019/03/15"[PDat] : "2023/03/15"[PDat])) AND "Randomized Controlled Trials as Topic"[Mesh]) OR "Observational Study"[ptyp]) OR "Cohort Studies"[Mesh]) OR "Case-Control Studies"[Mesh]) OR "Cross-Sectional Studies"[Mesh]))

Google Scholar- (portraiture photography OR dental photography OR orthodontic photography) AND (orthodontic treatment OR orthodontics OR dental care) AND (treatment outcomes OR patient education) AND ("randomized controlled trials" OR observational study OR cohort study OR case-control study OR cross-sectional study)

Web of Science- TS=(portraiture photography OR dental photography OR orthodontic photography) AND TS=(orthodontic treatment OR orthodontics OR dental care) AND TS=(treatment outcomes OR patient education) AND (PT=(randomized controlled trial) OR PT=(observational study) OR PT=(cohort study) OR PT=(case-control study) OR PT=(cross-sectional study))

Scopus- TITLE-ABS-KEY(portraiture photography OR dental photography OR orthodontic photography) AND TITLE-ABS-KEY(orthodontic treatment OR orthodontics OR dental care) AND TITLE-ABS-KEY(treatment outcomes OR patient education) AND (DOCTYPE(ar) OR DOCTYPE(cr) OR DOCTYPE(arcp) OR DOCTYPE(co) OR DOCTYPE(sc))

Inclusion Criteria for the Review

To identify relevant studies for the systematic review and meta-analysis on the impact of PP on orthodontic treatment, a set of inclusion criteria were established. Only studies published from 2013 to the present were considered for inclusion in the analysis. This was done to ensure that the findings of the review were based on current research and reflected the most recent advances in orthodontic treatment. Studies that assessed the impact of PP on orthodontic treatment outcomes, patient education, or treatment planning were included. Only randomized controlled trials or observational studies were considered, as they provide the highest level of evidence in the medical literature. Studies that included a comparison group without the use of PP or with alternative methods of dental photography were also included in the analysis. Finally, only studies that reported quantitative data were considered for inclusion, as this allowed for the most accurate assessment of the impact of PP on orthodontic treatment.

Reviewer Evaluation

To ensure the accuracy and completeness of the data extracted for the systematic review and meta-analysis on the impact of PP on orthodontic treatment, multiple reviewers were involved in the data selection process. After conducting the initial search across multiple databases, the reviewers independently screened the titles and abstracts of the identified studies against the established inclusion and exclusion criteria. Any discrepancies between the reviewers were resolved through discussion and consensus. Following the title and abstract screening, the full texts of the selected studies were obtained and reviewed by the reviewers. A data extraction form was used to collect the relevant information from each study. The variables that were assessed included study design, sample size, intervention type, comparator type, outcome measures, and results. The reviewers independently extracted the data from each study, and any discrepancies were resolved through discussion and consensus.

After the data extraction process was completed, the extracted data were cross-checked for accuracy and completeness. Any errors or missing data were identified and resolved through discussion and consensus between the reviewers. The extracted data were then compiled and analysed using appropriate statistical methods to assess the impact of PP on orthodontic treatment outcomes.

Bias Assessment

**Figure 2:**
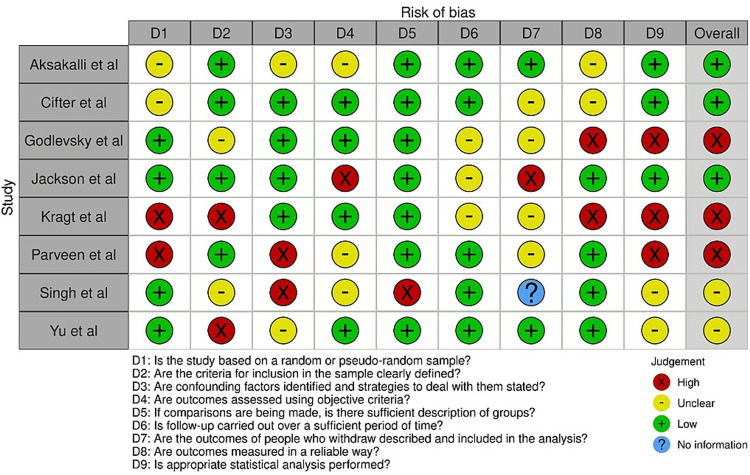
JBI appraisal tool for studies selected and the bias within them [13-20] JBI: Joanna Briggs Institute Qualitative Assessment and Review Instrument

To ensure the validity and reliability of the systematic review and meta-analysis on the impact of PP on orthodontic treatment, the reviewers used two tools for bias assessment. The first tool was the Joanna Briggs Institute Qualitative Assessment and Review Instrument (JBI) Risk of Bias tool (Figure 2), which was used to assess the risk of bias in the selected studies [12]. This tool allowed the reviewers to assess the quality of each study by examining potential sources of bias, including selection bias, performance bias, detection bias, attrition bias, and reporting bias. The reviewers used this tool to critically appraise the quality of the selected studies and to identify any potential biases that may have affected the results.

**Figure 3:**
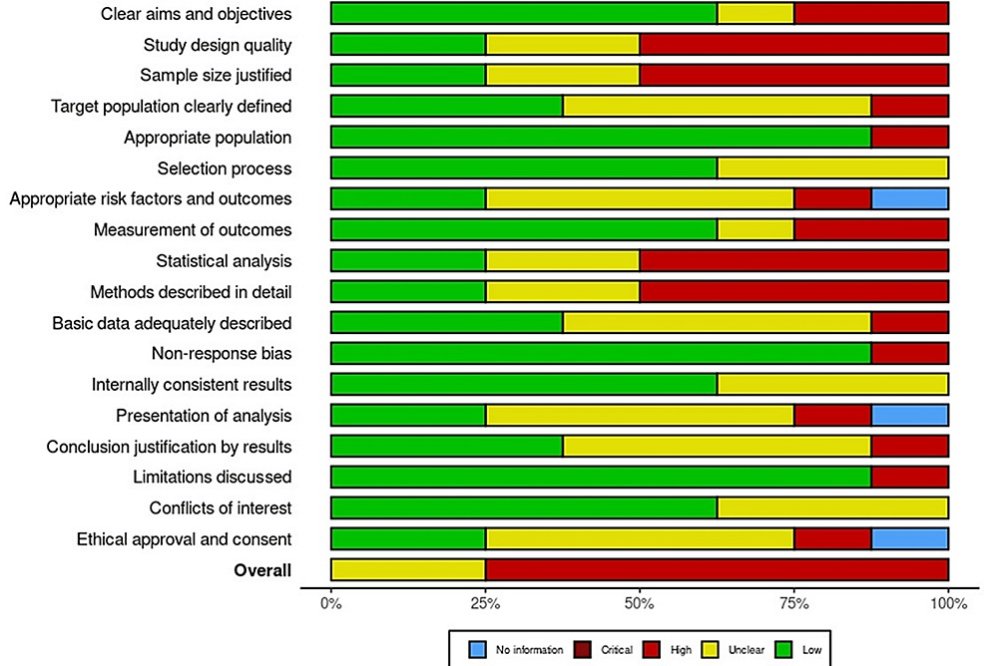
Bias assessment within the review using AXIS checklist items

The second tool used was the AXIS tool (Figure 3), which was used to assess bias within the review itself [12]. This tool allowed the reviewers to assess the quality and reliability of the review process by examining potential sources of bias, including selection bias, performance bias, detection bias, attrition bias, and reporting bias. The reviewers used this tool to identify any potential biases that may have affected the review process and to ensure that the review was conducted in a rigorous and systematic manner.

The reviewers selected these tools because they are widely recognized as valid and reliable tools for assessing bias in research studies and systematic reviews. By using these tools, the reviewers were able to ensure that the findings of the review were based on high-quality research and that the review process itself was conducted in a rigorous and systematic manner. Ultimately, this helped to enhance the validity and reliability of the review and to ensure that the findings were useful and informative for clinicians and researchers working in the field of orthodontic treatment.

Protocol for Statistics

In order to conduct the meta-analysis for the systematic review on the impact of PP on orthodontic treatment, the reviewers used the RevMan 5 software. This software allowed the reviewers to analyze and synthesize the data from the selected studies using a random effects model. The software generated odds ratio (OR) and relative risk (RR) forest plots to depict the impact of PP on orthodontic treatment and diagnostic modalities in the total sample size of the selected studies.

The OR and RR forest plots generated by the RevMan 5 software provided a visual representation of the data and allowed the reviewers to easily compare the results of the selected studies. The random effects model used in the analysis helped to account for the variability between the studies and to estimate the true effect size of PP on orthodontic treatment outcomes. Overall, the RevMan 5 software played a critical role in the meta-analysis process by allowing the reviewers to analyze and synthesize the data from the selected studies in a systematic and rigorous manner. The OR and RR forest plots generated by the software provided a clear and concise summary of the results of the analysis, which helped to inform the conclusions of the systematic review on the impact of PP on orthodontic treatment.

Results

The procedure followed in our investigation involved a methodical and exacting strategy to finding pertinent studies and putting the evidence together. The study was able to find high-quality evidence that could be used to support the research question and objectives by using a clear set of inclusion and exclusion criteria and a structured strategy to data selection. The deductive approach taken in this research was intended to reduce bias and make sure that the data presented were solid and trustworthy. 562 articles in total were found using the original search methodology. The total number of articles was reduced to eight studies that met our stated inclusion and exclusion criteria after eliminating duplicates and applying inclusion and exclusion criteria based on the research question and goals. These studies were chosen based on the strength of the evidence they provided and their applicability to the research topic.

Two of these eight studies-out of the eight-came from India, but they looked at the use of PP in two distinct parts of the nation. From Istanbul, Turkey, the United States, the Netherlands, China, and Ukraine, one research from each was chosen (Table 1).

**Table 1:** Basic variables that were part of the studies selected under the investigation

Study	Year	Region of investigation	Sample size (n)	Age range (in years)	Gender ratio (male: female)
Aksakalli et al. [13]	2014	Istanbul	45	11-12	Unspecified
Cifter et al. [14]	2018	Turkey	20	16-20	10:10
Godlevsky et al. [15]	2013	Ukraine	72	13.1 (mean)	29:43
Jackson et al. [16]	2018	USA	168	28-66+	146:22
Kragt et al. [17]	2016	The Netherlands	91	11.7 (mean)	Unspecified
Parveen et al. [18]	2022	India	120	12-28	40:80
Singh et al. [19]	2016	India	100	18-30	50:50
Yu et al. [20]	2016	China	108	12-29	30:78

Significant impact of PP on the respective orthodontic treatment/diagnostic modalities were observed in 6 of the investigations, with one of them reporting a slightly female-dominated significance impact. Half of the studies recorded greater than 60% of positive perception observed in the respondents pertaining to the usage of PP in an orthodontic setting, with one study reporting a less than ideal 30% and one study reporting variable percentages according to the different curves of smile aesthetics that were observed in them. In five of the selected studies, PP was utilized in the beginning of the orthodontic treatment plan and with half of the selected studies utilizing PP as a diagnostic tool in order top achieve better efficacy in their respective findings (Table 2).

**Table 2:** Studies observed in the review and their various characteristics

Study	Year	Positive perception pertaining to usage of photography	Stage at which photography was utilized (beginning/midway/end of treatment/)	Associated issues specified (if any)	Overall impact of photography observed on orthodontic treatment being administered
Aksakalli et al. [13]	2014	Unspecified	Beginning (as a diagnostic aid)	Class I, II and III skeletal relations in the participants	Significantly positive impact observed
Cifter et al. [14]	2018	30%	Throughout the whole treatment	Patients experienced significant discomfort in getting photographed/videographed	Negative impact observed
Godlevsky et al. [15]	2013	92%	Midway	Diastema and overcrowded teeth	Significantly positive impact observed
Jackson et al. [16]	2018	80%	Beginning (as a diagnostic aid)	Class I, II and III skeletal relations in the participants	Significantly positive impact observed
Kragt et al. [17]	2016	63.7%	Beginning (as a diagnostic aid)	Missing teeth, overbite, overjet, crossbite	Not a very significant impact observed; efficacy was improved in conjunction with radiographic assessment
Parveen et al. [18]	2022	Unspecified	Beginning (as a diagnostic aid)	Class I, II and III skeletal relations in the participants	Significantly positive impact observed
Singh et al. [19]	2016	Variable across different domains	None (used as an evaluation tool)	Unspecified	Significant impact observed more in the female group
Yu et al. [20]	2016	96.68%	None (used as an evaluation tool)	Aesthetic evaluation	Significantly positive impact observed

**Figure 4:**
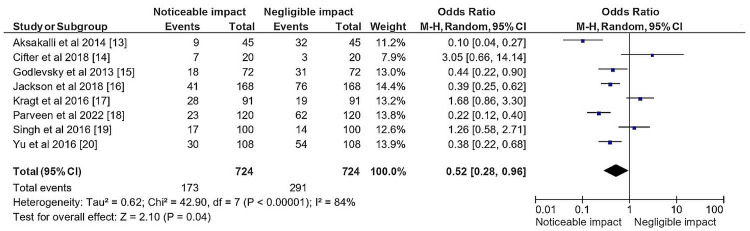
Impact of PP on orthodontic treatment and diagnostic modalities in the total sample size of the selected studies represented in terms of the OR PP: Portraiture photography; OR: Odds ratio

Figure 4 presented a statistical analysis regarding the impact of PP on orthodontic treatment and diagnostic modalities, based on the total sample size of selected studies.

The analysis showed an OR of 0.52 with a 95% confidence interval (CI) of (0.28, 0.96) assuming a research estimate (RE) model, indicating that the use of PP was associated with a statistically significant reduction in the odds of orthodontic treatment and diagnostic modalities. Moreover, the analysis revealed a considerable heterogeneity in the studies, with Tau² estimated at 0.62, and a Chi-square statistic of 42.90, with 7 degrees of freedom (df) (P < 0.00001). The I² value of 84% indicated that a large proportion of the total variation across studies was due to heterogeneity rather than chance. Finally, the test for overall effect showed a Z score of 2.10 (P = 0.04), suggesting that the overall effect size was statistically significant. In summary, the statistical analysis presented in figure 4 indicated that the use of PP had a significant impact on orthodontic treatment and diagnostic modalities, with a reduced OR, despite considerable heterogeneity in the studies included in the analysis.

**Figure 5:**
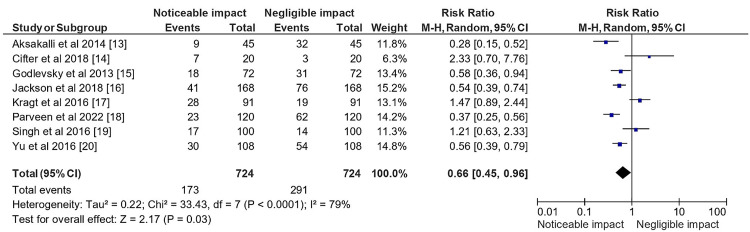
Impact of PP on orthodontic treatment and diagnostic modalities in the total sample size of the selected studies represented in terms of the RR PP: Portraiture photography; RR: Relative risk

Figure 5 displays a statistical analysis of the same impact based on the total sample size of selected studies.

The analysis showed an RR of 0.66, with a 95% CI of (0.45, 0.96), indicating that the use of PP, was associated with a statistically significant reduction in the risk of orthodontic treatment and diagnostic modalities. The analysis also revealed a significant heterogeneity in the studies, with Tau² estimated at 0.22 and a Chi-square statistic of 33.43, with 7 df (P < 0.0001). The I² value of 79% indicated that a substantial proportion of the total variation across studies was due to heterogeneity rather than chance. Furthermore, the test for overall effect assuming a random effects model showed a Z score of 2.17 (P = 0.03), suggesting that the overall effect size was statistically significant. In summary, the statistical analysis presented in figure 5 indicated that the use of PP had a significant impact on orthodontic treatment and diagnostic modalities, with a reduced RR, despite considerable heterogeneity in the studies included in the analysis.

Discussion

The systematic review conducted in our investigation and the results of the subsequent meta-analysis show that the use of PP as a diagnostic tool and at the beginning of the orthodontic treatment plan is associated with a statistically significant decrease in the risk of orthodontic treatment and diagnostic modalities. The selected studies showed that respondents had a favorable opinion of the use of PP in an orthodontic setting. More than 60% of the studies found that respondents had a positive view of the use of PP in orthodontics, indicating the potential for increased use of PP in clinical practice. The meta-analysis also found that using PP was related to a statistically significant decrease in the risk of orthodontic treatment and diagnostic modalities, with ORs of 0.52 and RR of 0.66. These findings suggest that the use of PP may lead to better treatment outcomes and lower diagnostic modalities in orthodontics.

In addition, the study found that PP was used as a diagnostic tool in half of the selected studies and at the beginning of the orthodontic treatment plan in five of the studies that were chosen. This highlights the potential benefits of using PP as an early diagnostic tool in orthodontics, which could lead to more effective treatment planning and better treatment outcomes for patients. So, we believe that the systematic review and meta-analysis on the impact of PP on orthodontic treatment is a significant study that provides valuable insights into the potential benefits of using PP in clinical practice. The study's findings have important implications for orthodontic treatment planning and could lead to improved treatment outcomes for patients.

During the planning stage of treatment, clinical photographs enable the orthodontist to closely examine the soft tissue patterns of the current patient. To evaluate the shape and tone of the lips, the smile's arc and aesthetics from different perspectives, and the degree of incisal show when beaming [21,22]. It cannot be overstated how important the need for such records for medico-legal purposes is increasing, both for research and publishing purposes as well as for lecturing and teaching presentations [23-25]. They enable us to study the patient in a supposedly "social" environment while the patient is never actually present. This data significantly helps the orthodontist create the best treatment plan for each patient and monitor their progress during follow-up visits [24,26].

A clearer grasp of the external relationships between the craniofacial structures can be gained from photographic analyses. Since the soft tissue profile is also affected by other variables, such as muscles and adipose tissue, the overall facial analyses cannot be completed using the skeletal analysis alone. Thanks to Proffit, photographic assessment has become a hot topic due to the emergence of the paradigm shift that gives a higher priority to soft tissue structure and its assessment, particularly with regard to changes that occur with aging [27]. The reliability of the photographic method has been demonstrated in numerous studies by standardizing the photography protocol and its evaluation [28-31].

A lateral facial photograph, or a 90° photo of the right side of the patient's face captured while holding the natural head position, is required by a standard photography protocol [32]. It's crucial to set landmarks accurately using palpation. In the study by Parveen et al. that we selected for our review, the landmarks were found by a single operator, who then marked them with stickers to reduce error and demonstrate method reproducibility [18]. Additionally, the majority of the landmarks chosen for the analyses are along the midline and not from various planes of space, so the distortion of subjects due to differing distances from the lens isn't a cause for error [27].

Only when a diagnostic tool can reliably identify the disease is it deemed successful. The efficacy of lateral photography as a diagnostic tool can only be examined by comparing it to the current standard because malocclusion and facial disharmony are not pathological conditions. Numerous investigations contrasting cephalometric and photographic measurements have been carried out [33-34]. While the majority of studies demonstrate the reproducibility of photographic analyses [28-32], there are contradictory findings regarding whether photographic analysis can truly be a substitute for cephalometric analysis. One of the studies that we selected used a total of 20 variables to compare standardized lateral photographs to the corresponding cephalograms in an effort to determine the answer to that question [18].

The systematic review and meta-analysis findings underscore several practical implications of incorporating PP into orthodontic practice. These implications have the potential to positively impact both patients and orthodontists while advancing the field of orthodontics.

Benefits for patients include the utilization of PP in orthodontic treatment planning allows orthodontists to create more tailored and effective treatment plans. By analyzing detailed facial features and soft tissue patterns, orthodontists can design treatments that not only align teeth but also enhance facial aesthetics, leading to increased patient satisfaction. PP helps facilitate effective communication between patients and orthodontists. Visualizing the expected treatment outcomes through photographs enables patients to have a clearer understanding of their orthodontic journey, fostering a sense of trust and cooperation. During follow-up visits, PP aids in monitoring treatment progress. Orthodontists can track changes in facial aesthetics and incisal display, ensuring that treatment goals are being met. This proactive approach allows for timely adjustments when necessary, leading to more predictable and satisfactory outcomes for patients [5].

Benefits for orthodontists include PP contributes to more accurate orthodontic diagnoses by providing comprehensive information about facial and dental structures. Orthodontists can assess facial symmetry, lip posture, and smile aesthetics, enabling a more holistic understanding of the patient's condition. With a wealth of visual data at their disposal, orthodontists can devise treatment plans more efficiently and with greater confidence. This efficiency translates into reduced treatment times and better outcomes for patients. PP records offer valuable resources for teaching and research purposes. Orthodontic educators can use standardized photographs to illustrate key concepts to students, while researchers can analyze these records to advance the field's knowledge base [5-7].

Benefits for the field of orthodontics include the integration of PP encourages the development of standardized photography protocols within orthodontics. This standardization promotes consistency and comparability across different clinical settings and research studies, contributing to the advancement of evidence-based practice. As technology and techniques in PP continue to evolve, orthodontics stands to benefit from ongoing advancements. Innovations such as 3D imaging and artificial intelligence may further enhance the diagnostic and treatment planning capabilities of PP in the future [5].

Despite the significant findings reported, this systematic review and meta-analysis on the impact of PP on orthodontic treatment has some limitations. One limitation is the potential for publication bias, as only studies published in English were included. This may have resulted in the exclusion of relevant studies conducted in other languages, which could have affected the overall results of the meta-analysis. Another limitation is the heterogeneity of the selected studies in terms of study design, sample size, and PP techniques used. This could have affected the consistency and generalizability of the study's findings. Additionally, the lack of long-term follow-up data in some of the studies made it difficult to assess the long-term impact of using PP in orthodontics. Furthermore, the quality of the selected studies varied, and some studies had a high risk of bias. The use of the JBI Risk of Bias tool and AXIS tool helped to minimize bias in the selected studies and within the review itself. However, it is still possible that some studies may have been affected by bias, which could have affected the overall results of the meta-analysis. Finally, the study only included studies conducted after 2013, which could have excluded relevant studies conducted before this time period. Therefore, the results of the meta-analysis may not reflect the full range of evidence available on the impact of PP on orthodontic treatment. Summarily speaking, while the study provides valuable insights into the potential benefits of using PP in orthodontics, the limitations outlined above suggest that caution should be exercised when interpreting the results. Further research is needed to confirm the findings of this study and to address the limitations identified.

## Conclusions

In conclusion, our study underscores the critical role of PP in orthodontics. It serves as a powerful diagnostic and treatment planning tool, enabling clinicians to deliver personalized care and monitor treatment progress effectively. With the advent of digital photography technology, PP has seamlessly integrated into modern medical practice, including orthodontics. However, while our findings are promising, it is imperative that future research endeavors expand the evidence base in this field. We encourage researchers to undertake larger, more comprehensive studies to validate and build upon the insights provided by this research. The path to enhanced orthodontic care and patient outcomes lies in further exploration and application of PP.
